# Simple steatosis sensitizes cholestatic rats to liver injury and dysregulates bile salt synthesis and transport

**DOI:** 10.1038/srep31829

**Published:** 2016-08-18

**Authors:** Daniël A. Lionarons, Michal Heger, Rowan F. van Golen, Lindy K. Alles, Vincent A. van der Mark, Jaap J. Kloek, Dirk R. de Waart, Hendrik A. Marsman, Henny Rusch, Joanne Verheij, Ulrich Beuers, Coen C. Paulusma, Thomas M. van Gulik

**Affiliations:** 1 Department of Experimental Surgery, Academic Medical Center, University of Amsterdam, Amsterdam, the Netherlands; 2 Tytgat Institute for Liver and Intestinal Research, Academic Medical Center, University of Amsterdam, Amsterdam, the Netherlands; 3 Laboratory Genetic Metabolic Diseases, Academic Medical Center, University of Amsterdam, Amsterdam, the Netherlands; 4 Department of Pathology, Academic Medical Center, University of Amsterdam, Amsterdam, the Netherlands

## Abstract

Nonalcoholic fatty liver disease (NAFLD) is the most common chronic liver disorder. It is uncertain if simple steatosis, the initial and prevailing form of NAFLD, sensitizes the liver to cholestasis. Here, we compared the effects of obstructive cholestasis in rats with a normal liver versus rats with simple steatosis induced by a methionine/choline-deficient diet. We found that plasma liver enzymes were higher and hepatic neutrophil influx, inflammation, and fibrosis were more pronounced in animals with combined steatosis and cholestasis compared to cholestasis alone. Circulating bile salt levels were markedly increased and hepatic bile salt composition shifted from hydrophilic tauro-β-muricholate to hydrophobic taurocholate. This shift was cytotoxic for HepG2 hepatoma cells. Gene expression analysis revealed induction of the rate-limiting enzyme in bile salt synthesis, cytochrome P450 7a1 (CYP7A1), and modulation of the hepatic bile salt transport system. In conclusion, simple steatosis sensitizes the liver to cholestatic injury, inflammation, and fibrosis in part due to a cytotoxic shift in bile salt composition. Plasma bile salt levels were elevated, linked to dysregulation of bile salt synthesis and enhanced trafficking of bile salts from the liver to the systemic circulation.

Nonalcoholic fatty liver disease (NAFLD) is the most common chronic liver disorder^1^. Prevalence is worldwide estimated at 20%, and reported up to 46% in Western populations^1^. NAFLD is characterized by the accumulation and vesicularization of triglycerides in hepatocytes. The condition encompasses a pathological spectrum from simple steatosis to nonalcoholic steatohepatitis (NASH), fibrosis, cirrhosis, and ultimately end-stage liver disease or hepatocellular carcinoma^1^. The pathogenesis and disease progression have been studied extensively but are still poorly understood. NAFLD is closely associated with obesity, dyslipidemia, and diabetes mellitus type 2^2^. Together with these disorders, the prevalence of NAFLD is expected to rise in the coming decades, and NAFLD is projected to become the primary indication for liver transplantation^2^.

It is estimated that 75–90% of NAFLD patients have only simple steatosis^3^, considered to be a benign stage without the need for intervention^1^,^4^. However, steatotic livers are unable to tolerate a variety of challenges that normal livers can effectively cope with. Simple steatosis sensitizes the liver to injury by toxins^5^ and postischemic reperfusion^6^, decreases the regenerative capacity of the liver^7^, and increases the incidence of complications following liver transplantation^6^. In addition, studies have shown that patients and animals with fatty livers have elevated plasma bile salt levels^8^,^9^,^10^,^11^,^12^, suggesting that NAFLD disturbs bile salt homeostasis. However, whether simple steatosis sensitizes the liver to injury from obstructive cholestasis is currently elusive, as is the influence of combined steatosis and cholestasis on bile salt homeostasis.

Therefore, we investigated the effect of acute obstructive cholestasis on liver injury, inflammation, and bile salt homeostasis in rats with simple hepatic steatosis versus rats without parenchymal liver disease. A 3-week methionine/choline-deficient (MCD) diet was employed to induce simple steatosis. In the third week, animals underwent common bile duct ligation (BDL) to induce obstructive cholestasis. The key finding is that simple steatosis aggravates cholestatic liver injury, inflammation, and fibrosis in association with altered bile salt synthesis and transport.

## Results

### Simple steatosis aggravates cholestatic liver injury and weight loss

**Figure 1:**
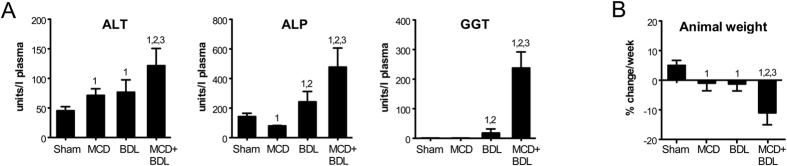
Liver injury and weight loss are increased in animals with combined steatosis and cholestasis compared to cholestasis alone. (**A**) Plasma levels of alanine transaminase (ALT), alkaline phosphatase (ALP), and gamma-glutamyl transferase (GGT). (**B**) Animal weight change during the third week of the experiment, starting directly before sham surgery or BDL. Data represent means ± SD of *n* = 5–6/group; 1, *p* < 0.05 versus sham; 2, *p* < 0.05 versus MCD; 3, *p* < 0.05 versus BDL.

Plasma alanine transaminase (ALT), alkaline phosphatase (ALP), and gamma-glutamyl transferase (GGT) were increased 1.6-fold (*p* < 0.05), 2.0-fold (*p* < 0.05), and 13.0-fold (*p* < 0.001), respectively, in animals with combined steatosis and cholestasis versus cholestasis alone (Fig. 1A). The synthetic function of the liver was largely similar in these groups, as evidenced by similar prothrombin times and plasma levels of albumin and fibrinogen (Supplementary Fig. S2). Only plasma antithrombin levels were lower in both steatosis and combined steatosis and cholestasis versus cholestasis (*p* < 0.01). A striking finding was that animals with combined steatosis and cholestasis were unable to maintain their weight, exhibiting an 11.0% reduction in body weight during the week they were subjected to BDL (Fig. 1B). Steatotic and cholestatic rats lost only 1.0% and 1.3% body weight, respectively (*p* < 0.001 versus combined steatosis and cholestasis). Thus, acute obstructive cholestasis induced more extensive liver injury and weight loss in steatotic than in normal animals.

### Cholestatic inflammation and fibrosis are increased in steatotic livers compared to normal livers

**Figure 2:**
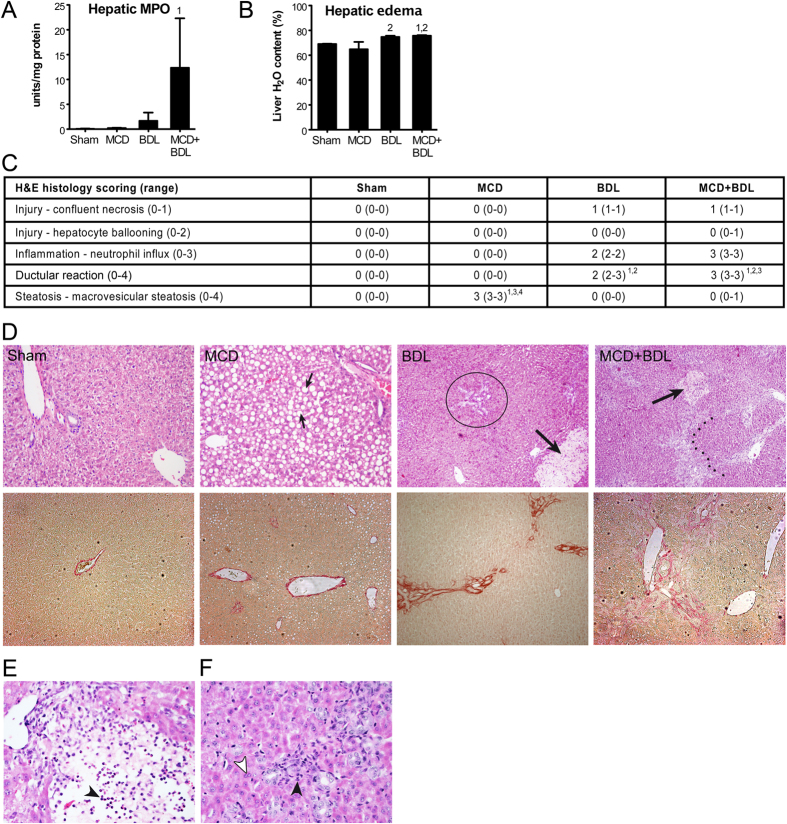
Cholestasis-induced inflammation and fibrosis are exacerbated in livers of animals with simple steatosis. (**A**) Hepatic MPO activity. (**B**) Hepatic edema. (**C**) Semi-quantitative analysis of H&E-stained sections. Statistical analysis was not performed on scores for confluent necrosis and neutrophil influx because ranges were confined to one value. (**D**) Representative liver histology. Upper panels, H&E staining; lower panels, picrosirius red staining; small arrows, macrovesicular steatosis; encircled, discrete periportal ductular reaction; large arrows, confluent necrosis; dotted line, porto-portal septal fibrosis. Neutrophil influx was associated with confluent necrosis (**E**) and ductular reaction (**F**). Black arrowheads, nucleus of inflammatory cell; white arrowhead, nucleus of hepatocyte. Values represent *n* = 5–6/group; histological scoring data are reported as median (range), other data as means ± SD; 1, *p* < 0.05 versus sham; 2, *p* < 0.05 versus MCD; 3, *p* < 0.05 versus BDL; 4, *p* < 0.05 versus MCD+BDL.

The concentration of pro-inflammatory cytokines tumor necrosis factor α (TNF-α) and interleukin 6 (IL-6) were determined in liver homogenates. No significant differences between steatosis, cholestasis, and combined steatosis and cholestasis were detected (Supplementary Fig. S3). However, hepatic myeloperoxidase (MPO) activity, a measure of the degree of neutrophil influx, tended to be higher (7.4-fold) in animals with combined steatosis and cholestasis in comparison to cholestasis (Fig. 2A). The degree of hepatic edema was aggravated by cholestasis and combined steatosis and cholestasis but not by steatosis alone (Fig. 2B). Histological scoring demonstrated that the extent of macrovesicular steatosis was consistently >66% in steatotic livers but <33% when steatosis and cholestasis were combined (*p* < 0.001, Fig. 2C,D). Of note, the NAFLD activity score indicated that none of the animals met the histological criteria for NASH (Supplementary Table S1). The MPO data were confirmed histologically, as neutrophil influx was higher in the liver of animals with combined steatosis and cholestasis compared to cholestatic livers. The influx of neutrophils was associated with areas of confluent necrosis (Fig. 2E) and ductular reaction (Fig. 2F). Septal fibrosis was consistently observed in livers with combined steatosis and cholestasis (5/5 animals) but not in cholestatic livers (2/6 animals), which mainly exhibited periportal fibrosis without septum formation (4/6 animals, *p* < 0.01). Thus, acute obstructive cholestasis induced a stronger inflammatory and fibrotic reaction in steatotic compared to normal livers.

Reactive oxygen species (ROS) are thought to be a pathogenic factor in both steatosis and cholestasis^13^,^14^. Excessive neutrophil influx and consequent MPO release could be a source of ROS, which may stimulate disease progression and further neutrophil influx in animals with combined steatosis and cholestasis^14^. To explore this possibility, we assessed the hepatic antioxidant capacity, which reflects the ability of the liver to neutralize ROS and is suppressed by prolonged and severe oxidative stress. However, there were no differences observed between cholestasis and combined steatosis and cholestasis (Supplementary Fig. S4), suggesting that oxidative stress does not drive differences in liver injury and inflammation.

### Increased plasma bile products and a more cytotoxic bile salt composition in animals with combined steatosis and cholestasis versus cholestasis alone

**Figure 3:**
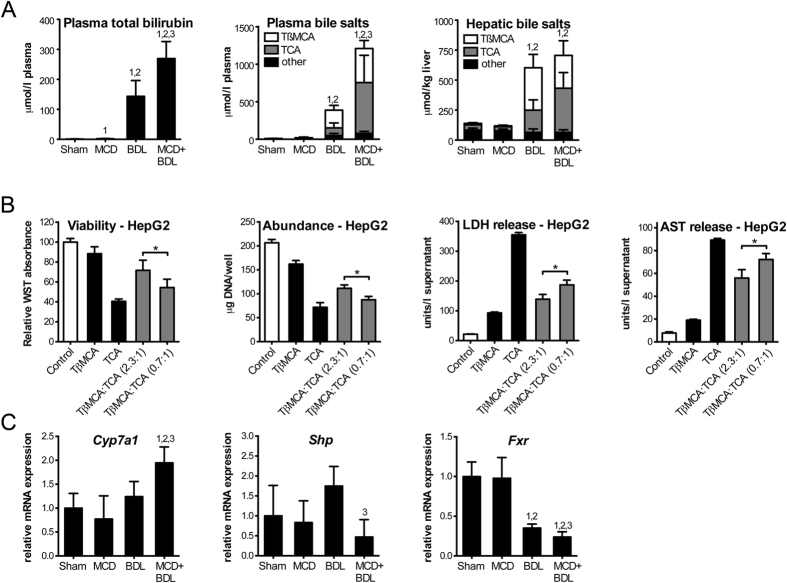
Dysregulation of bile salt synthesis in animals with combined steatosis and cholestasis. (**A**) Plasma total bilirubin, plasma bile salt levels, and hepatic bile salt levels. Statistical analyses shown were performed on total bile salt levels. (**B**) HepG2-NTCP cells treated with bile salts at 200 μmol/L in total were assayed for viability, abundance, and release of AST and LDH. (**C**) Hepatic mRNA of *Cyp7a1* and nuclear receptors that suppress *Cyp7a1*. Values represent means (*n* = 4–6) ± SD; 1, *p* < 0.05 versus sham; 2, *p* < 0.05 versus MCD; 3, *p* < 0.05 versus BDL; *p < 0.05.

Plasma total bilirubin was 144 ± 53 μmol/L in cholestatic rats versus 270 ± 56 μmol/L in rats with combined steatosis and cholestasis (*p* < 0.05), and plasma total bile salt levels were 388 ± 89 μmol/L in cholestatic rats versus 1,210 ± 395 μmol/L in rats with combined steatosis and cholestasis (*p* < 0.001, Fig. 3A). Plasma total bile salt measurements performed by high-performance liquid chromatography (HPLC) were consistent with results from an enzyme cycling assay (Supplementary Fig. S5).

The marked increase in plasma bile products in animals with combined steatosis and cholestasis compared to cholestasis prompted an investigation of the underlying mechanisms. First, hepatic total bile salts were determined. In contrast to plasma total bile salts, levels in liver homogenates were comparable, namely 604 ± 98 μmol/L in cholestasis versus 706 ± 260 μmol/L in combined steatosis and cholestasis (*p* > 0.05, Fig. 3A). Next, the composition of the bile salt pool was assessed. Tauro-β-muricholate (TβMCA) and taurocholate (TCA) accounted for 85–95% of total bile salts in the plasma and the liver of animals with cholestasis and combined steatosis and cholestasis. However, animals with combined steatosis and cholestasis exhibited a balance shift in that the relative abundance of TCA became predominant at the expense of TβMCA. The TβMCA/TCA ratio in plasma was 3.1 ± 1.8 in cholestatic animals compared to 0.9 ± 0.7 in animals with combined steatosis and cholestasis (*p* < 0.01). Similarly, the hepatic TβMCA/TCA ratio was 2.3 ± 1.2 and 0.7 ± 0.2 in cholestasis versus combined steatosis and cholestasis, respectively (*p* < 0.05). Taken together, pre-existent simple steatosis caused extensive dysregulation of bile salt homeostasis in bile duct-ligated rats.

To test if the differential bile salt composition could have played a role in the exacerbation of liver injury in animals with combined steatosis and cholestasis, we assessed hepatocellular toxicity resulting from treatment with TβMCA and TCA in HepG2-NTCP cells. Cell viability and total DNA (i.e., cell number) were decreased and release of aspartate transaminase (AST) and lactate dehydrogenase (LDH) was increased when cells were treated with TβMCA/TCA at a ratio of 0.7 (as observed in the liver of animals with combined steatosis and cholestasis), when compared to a ratio of 2.3 (as observed in the liver of animals with cholestasis, *p* < 0.05, Fig. 3B). These results indicate that the observed reduction in the TβMCA/TCA ratio is sufficient to increase hepatocellular toxicity, while the total bile salt level remains unchanged.

### Simple steatosis dysregulates bile salt synthesis in cholestatic rats

Next, we quantified hepatic gene expression of cytochrome P450 7a1 (CYP7A1), the rate-limiting enzyme in the conversion of cholesterol into bile salts^15^. *Cyp7a1* mRNA was more abundant in animals with combined steatosis and cholestasis compared to other experimental groups (*p* < 0.05, Fig. 3C). *Cyp7a1* is tightly regulated by a feedback repression cascade involving the nuclear receptors farnesoid X receptor (FXR/*Nr1h4*) and small heterodimer partner (SHP/*Nr0b2*)^15^,^16^,^17^. Bile salts are endogenous ligands of FXR, which upon activation binds to the SHP promoter to initiate transcription. SHP then represses transcription of *Cyp7a1*. Hepatic mRNA levels of *Shp* and *Fxr* in animals with steatosis and cholestasis decreased to 27% (*p* < 0.01) and 69% (*p* < 0.05) of the levels in cholestatic animals, respectively. In sum, gene expression analysis suggests that bile salt production is promoted through dysregulation of feedback repression.

Bile salt species have differential potencies in activating FXR, and the relief of feedback repression may have occurred through alterations in the bile salt composition. We did not detect differences in *CYP7A1* and *SHP* mRNA when HepG2-NTCP cells were treated with TβMCA/TCA at a ratio of 2.3 versus 0.7 (similar to the ratio in the liver of animals with cholestasis and combined steatosis and cholestasis, respectively; *p* > 0.05; Supplementary Fig. S6). However, changes in less abundant but more potent FXR ligands could have played a significant role in the induction of *Cyp7a1*. The order of FXR activating potency of bile salts/acids is chenodeoxycholate (CDCA) > litocholate (LCA) = deoxycholate (DCA) > cholate (CA)^17^. Detailed analysis of the bile salt composition demonstrated that the concentration of unconjugated and conjugated CDCA, LCA, and DCA was generally similar or slightly elevated in the plasma and liver of animals with combined steatosis and cholestasis compared to cholestasis alone (Tables 1 and 2). These data suggest that changes in bile salt composition are not responsible for the dysregulation of *Cyp7a1*.

### Steatosis modulates gene expression of bile salt transporters in cholestatic rats

An increased production of bile salts may translate to increased bile salt levels in the systemic circulation while hepatic levels remain unchanged. We hypothesized that this phenomenon may result from altered transport across the basolateral membrane rather than canalicular efflux in the model of bile duct ligation. Therefore, the expression of bile salt and organic anion transporters in hepatocytes was studied.

**Figure 4:**
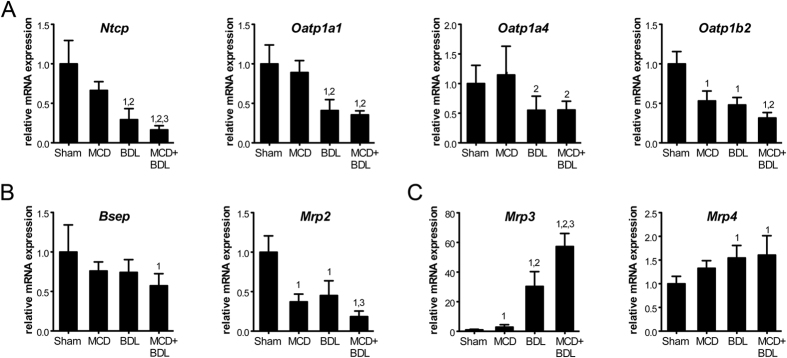
Gene expression of hepatic bile salt and bilirubin transporters is modulated in animals with combined steatosis and cholestasis. (**A**) Hepatic mRNA of basolateral transporters that import bile salts and bilirubin from the circulation. (**B**) Hepatic mRNA of canalicular transporters that excrete bile salts and bilirubin into the biliary system. (**C**) Hepatic mRNA of basolateral transporters that export bile salts and bilirubin into the circulation. Values represent means (*n* = 5–6) ± SD; 1, *p* < 0.05 versus sham; 2, *p* < 0.05 versus MCD; 3, *p* < 0.05 versus BDL.

Circulating and hepatic bile salt levels are regulated by the concerted actions of the bile salt synthesis machinery and bile salt transporters^18^,^19^. Bile salts are primarily taken up from the portal circulation by basolateral membrane-localized Na^+^-taurocholate cotransporting polypeptide (NTCP/*Slc10a1*). In addition, members of the organic anion transporting polypeptide (OATP) family transport conjugated and unconjugated bile salts and bilirubin conjugates across the basolateral membrane. In rats, these consist of OATP1A1/*Slco1a1*, OATP1A4/*Slco1a4,* and OATP1B2/*Slco1b2*. These transporters are downregulated transcriptionally during cholestasis, primarily through signaling pathways activated by inflammatory cytokines and bile salts^18^,^20^. Accordingly, decreased mRNA levels were observed in cholestasis compared to sham (Fig. 4A). Pre-existent steatosis exacerbated the cholestasis-induced transcriptional suppression of *Ntcp* to 16% of control levels compared with 29% in animals with cholestasis alone (*p* < 0.05). In addition, we determined hepatic mRNA levels of bile salt exporters located at the canalicular membrane. Levels of the bile salt export pump (BSEP/*Abcb11)* were not decreased in combined steatosis and cholestasis compared to cholestasis, while levels of multidrug resistance-associated protein 2 (MRP2/*Abcc2)* were (*p* < 0.05, Fig. 4B).

The basolateral membrane of hepatocytes also contains MRPs that facilitate the efflux of bile salts and bilirubin. These are normally expressed at very low levels, but undergo marked upregulation as part of an adaptive response to cholestasis^18^,^19^,^20^. Accordingly, mRNA levels of MRP3/*Abcc3* and MRP4/*Abcc4* were increased in cholestatic versus control livers (*p* < 0.01, Fig. 4C). Moreover, *Mrp3* levels in livers with simple steatosis were 2.9-fold higher than in control livers (*p* < 0.001), and levels in livers with combined steatosis and cholestasis were 1.7-fold higher than in cholestatic livers (*p* < 0.05).

In summary, transporter gene expression was changed in animals with combined steatosis and cholestasis compared to cholestasis alone, supporting lowered influx and increased efflux of bile products across the basolateral membrane of hepatocytes.

## Discussion

In this study, we investigated whether simple steatosis sensitizes the liver to injury from obstructive cholestasis. An *in vivo* rat model was used that combines the MCD diet to induce simple steatosis and BDL to induce obstructive cholestasis. Our results indicate that pre-existent steatosis exacerbates hepatocellular damage from a cholestatic insult and augments the degree of hepatic inflammation and fibrosis.

A short-term MCD diet was applied in rats as a model for simple steatosis, which does not allow for progression to NASH^21^,^22^. Although histological scoring confirmed the absence of NASH (Supplementary Table S1), this model is associated with a low degree or early hepatic inflammation, as evidenced by elevated hepatic pro-inflammatory cytokine levels (Supplementary Fig. S3). Similarly, pro-inflammatory cytokines are increased in the serum of patients with simple steatosis^23^,^24^. The MCD model also reproduces the liver pathology seen in NAFLD patients, but a limitation is the associated weight loss and lack of systemic metabolic syndrome, which is incongruous with the human situation^25^. Consequently, our results should be contextualized by metabolic follow-up studies.

Notably, a marked decrease in macrovesicular steatosis was observed in animals with combined steatosis and cholestasis versus steatosis alone. The cholestasis-induced reduction in steatosis could result from a combination of factors. First, the lack of bile in the intestinal lumen will lead to impaired uptake of dietary lipids. Second, the substantially higher rate of weight loss in animals with combined steatosis and cholestasis may have been accompanied by an increase in β-oxidation of hepatic triglyceride reserves^26^. Finally, elevated circulating bile salt levels could have stimulated hepatic metabolism through binding to TGR5, a peripherally expressed bile salt-activated G protein-coupled receptor that stimulates energy expenditure. TGR5 agonists are reported to reduce hepatic steatosis in mice fed a high-fat diet^27^, and male *Tgr5* knockout mice on a high-fat diet showed increased development of steatosis^28^.

The aggravation of cholestatic liver injury in steatotic versus control livers in the presence of comparable hepatic bile salt levels may result from altered bile salt composition. In both animals with cholestasis and combined steatosis and cholestasis, the bile salt spectrum was dominated by TβMCA and TCA. However, the hepatic TβMCA/TCA ratio was 2.3 in cholestasis versus 0.7 in combined steatosis and cholestasis. We demonstrated that this fall in the TβMCA/TCA ratio is sufficient to increase cell death in HepG2 cells. The enzymatic pathway that synthesizes TβMCA has not been completely characterized, and we were unable to determine the molecular mechanism underlying the differential TβMCA/TCA ratio. TβMCA is an unusual bile salt in the sense that the 7-OH group is oriented in β-configuration, similar to ursodeoxycholate (UDCA), currently the only effective drug for the treatment of cholestatic disorders^29^,^30^. The only difference between taurine-conjugated UDCA (TUDCA) and TβMCA is that TβMCA contains a third -OH group at the C-6 position. This makes TβMCA more hydrophilic than TUDCA and TCA^31^. Hydrophobic bile salts are primarily responsible for liver injury in cholestasis through permeabilizing mitochondria and activating cell death and inflammatory pathways. A reduction in the hydrophobicity of the bile salt pool is thought to be one of the mechanisms by which UDCA alleviates cholestatic liver injury^29^. Because of the hydrophilicity and the structural resemblance to UDCA, there has been an interest in applying TβMCA for the treatment of cholestasis. TβMCA has been shown to protect against tauro- and glyco-CDCA-induced hepatocelullar injury in a manner quantitatively similar to TUDCA^32^,^33^,^34^, reportedly through activation of anti-apoptotic pathways^34^. Furthermore, apoptosis induced by the free fatty acid palmitate, a model for lipoapoptosis in NAFLD, was reduced when HepG2 cells were co-treated with TβMCA^34^. Our results and the reports from others indicate that increased liver injury in combined steatosis and cholestasis in part resulted from a reduction in the TβMCA/TCA ratio.

Of note, there are differences in the bile salt composition of rodents in comparison to humans. The rodent bile salt pool is dominated by CA and MCA conjugates, whereas the more hydrophobic human bile salt pool consists primarily of CDCA, CA, and DCA conjugates^15^,^35^. It is currently unclear if the human bile salt pool also becomes more cytotoxic when exposed to combined steatosis and cholestasis.

Besides the changes in bile salt composition, we observed a marked increase in the concentration of total bile salts and bilirubin in the plasma of animals with combined steatosis and cholestasis. Notably, there have been reports of elevated plasma bile salts in multiple NAFLD animal models and in patients with simple steatosis and NASH^8^,^9^,^10^,^11^,^12^. We decided to investigate the mechanism by which steatosis interferes with bile salt homeostasis in our model. Gene expression of the rate-limiting enzyme in bile salt synthesis, CYP7A1, was upregulated in the liver of animals with combined steatosis and cholestasis compared to cholestasis alone. Interestingly, Bechmann *et al.* reported that *CYP7A1* mRNA is elevated in obese patients with NASH and in steatotic HepG2 cells^8^. Taken together, these results suggest that steatosis promotes bile salt synthesis by dysregulating CYP7A1.

*Cyp7a1* is thought to be controlled in part through hepatic FXR activation and subsequent transcription of *Shp*, and in part through intestinal FXR activation and subsequent enterohepatic FGF15 signaling^15^. Because there are no luminal bile salts that activate intestinal FXR in both cholestasis and combined steatosis and cholestasis, we focused on the hepatic FXR/SHP pathway in search of potential differentiating factors. Hepatic *Shp* was downregulated in animals with combined steatosis and cholestasis compared to cholestasis alone, similar to observations by Bechmann *et al.* in obese patients with NASH^8^, suggesting that steatosis induces CYP7A1 via suppression of *Shp* mRNA. In addition, we quantified *Fxr* mRNA, which was decreased. However, the quantity of *Fxr* mRNA does not necessarily correspond with activation, and the role of *Fxr* in the dysregulation of *Cyp7a1* remains unclear from these data.

Elevated plasma bile salt levels in combination with increased *Cyp7a1* transcription and unaffected hepatic bile salt levels in the context of an obstructed biliary system suggest that the bile salt transport system is modulated to promote trafficking from the liver to the systemic circulation. Indeed, gene expression of the canalicular bile salt and bilirubin exporter *Mrp2* and basolateral bile salt importer *Ntcp* was decreased. In addition, gene expression of the basolateral bile salt and bilirubin exporter *Mrp3* was increased. These data are supported by decreased MRP2, decreased *Ntcp/*NTCP, and increased *Mrp3/*MRP3 expression levels in NAFLD animal models and patients^11^,^12^,^36^,^37^.

**Figure 5:**
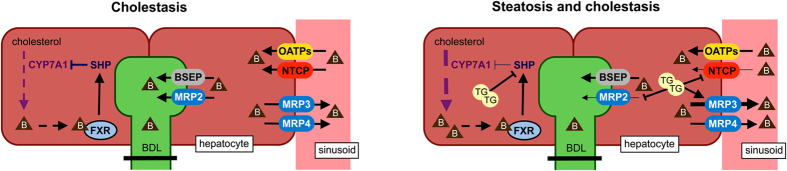
Working model that can explain why systemic bile salt levels are elevated in animals with combined steatosis and cholestasis. In response to obstructive cholestasis, SHP is transcriptionally suppressed in steatotic but not in normal hepatocytes. This relieves feedback repression of the rate-limiting enzyme in bile salt synthesis, CYP7A1. The subsequent increase in hepatic bile salts is countered by enhanced bile salt trafficking from the liver to the systemic circulation through downregulation of the canalicular exporter MRP2, downregulation of the basolateral importer NTCP, and upregulation of the basolateral exporter MRP3. Arrow weight changes represent differences between groups; arrows do not represent direct interactions; B, bile salts; TG, triglyceride droplets (i.e., steatosis).

On the basis of our data, we propose a working model in which bile salt synthesis is increased in steatotic hepatocytes undergoing a cholestatic insult, through transcriptional induction of *Cyp7a1*, via suppression of *Shp* (Fig. 5). Still, bile salts in the hepatic compartment are not increased due to modulation of the hepatocellular transport system. Influx of bile salts is decreased as a result of downregulated *Ntcp* expression, whereas efflux is increased through upregulated *Mrp3* expression. In addition, excretion into the (obstructed) canaliculus is suppressed through downregulation of *Mrp2* expression, further shifting bile salts from the hepatobiliary compartment to the systemic circulation.

In conclusion, simple steatosis induced by an MCD diet sensitized rats to cholestatic liver injury, inflammation, and fibrosis, in part due to increased cytotoxicity of the bile salt pool, in association with dysregulation of bile salt synthesis and transport.

## Methods

### Animals

All animal experiments were approved by the animal ethics committee at the Academic Medical Center in Amsterdam, the Netherlands (protocol BEX101653) and performed in accordance with Dutch guidelines and regulations. Male Wistar rats weighing 300 to 325 g were obtained from Harlan (Horst, the Netherlands). Animals were housed under standardized laboratory conditions that included *ad libitum* access to water and regular chow (Hope Farms, Woerden, the Netherlands), temperature and humidity control, and a 12-hour light/dark cycle. Upon arrival the animals were acclimatized for 1 week.

### Experimental design

Animals were fed regular chow or an MCD diet (Harlan Teklad, Madison, WI, USA) for 21 days to induce hepatic steatosis^38^. At day 14, all animals underwent either a sham operation or BDL (ref. 13 and supplementary methods). Cholestasis was maintained from day 14 to day 21, after which the animals were euthanized. The study was divided into 4 groups: sham, MCD, BDL, and MCD+BDL (*n* = 5–6/group).

### Histological analysis

Liver sections were stained with hematoxylin and eosin (H&E) or picrosirius red using routine clinical methods at the Department of Pathology of the Academic Medical Center, Amsterdam, the Netherlands^38^. An experienced hepatopathologist (J.V.) blinded to the groups analyzed the H&E-stained sections in a semi-quantitative fashion. Steatosis, lobular inflammation, and hepatocellular ballooning were assessed according to the NAFLD activity scoring system^39^. Confluent necrosis was determined as either absent (0) or present (1). Neutrophil influx was scored as absent (0), sparse (1), moderate (2), or pronounced (3). Reactive neutrophil accumulation due to surgery was excluded from the analysis. Ductular reaction, inflammation, and fibrosis were scored as absent (0), portal (1), periportal (2), septal (3), or cirrhosis (4).

### Biochemical analysis

Blood plasma or cell culture medium was assayed for ALT, AST, ALP, GGT, LDH, total bilirubin, albumin, prothrombin time, fibrinogen, and antithrombin using routine clinical chemistry at the Department of Clinical Chemistry of the Academic Medical Center, Amsterdam, the Netherlands. MPO activity, TNF-α, IL-6, and the total antioxidant capacity were measured in liver homogenates as described previously^13^,^40^. Total bile salt levels were determined with an enzyme cycling assay (Diazyme Laboratories, Poway, CA, USA) using a NOVOstar microplate reader (BMG Labtech, Offenburg, Germany). Bile salt composition was assessed by reverse-phase HPLC^35^, as described in the supplementary methods.

### Bile salt toxicity assay

HepG2 human hepatoma cells stably expressing rat NTCP^41^ were cultured at 37 °C in humidified air with 5% CO_2_ in phenol red-lacking Williams E medium (Lonza, Basel, Switzerland) supplemented with 10% FCS (Bodinco, Alkmaar, the Netherlands), 5 μg/mL insulin (Sigma, Saint Louis, MO, USA), 50 μmol/L hydrocortisone hemisuccinate (Sigma), 100 IU/mL penicillin (Lonza), 0.1 mg/mL streptomycin (Lonza), and 2 mmol/L L-glutamine (Lonza). Cells were seeded in 24-well plates. When reaching ~80% confluence, cells were treated with 200 μmol/L bile salts, consisting of TβMCA (Steraloids, Newport, RI, USA) and/or TCA (Sigma) in a 1:0, 0:1, 2.3:1, or 0.7:1 molar ratio. After 24 h of incubation, the cell culture supernatant was collected for measurement of LDH and AST.

To measure cell viability, the monolayer was washed twice with PBS and the cells received 310 μL of culture medium containing 10 μL of WST-1 reagent (Roche Applied Science, Penzberg, Germany). After 10 min of incubation, 200 μL of medium from each well was transferred to a 96-well plate and the absorbance was read at 450 nm on a Synergy HT microplate reader (BioTek, Winooski, VT, USA).

To measure DNA content (i.e., the number of cells) per well, the cells were washed with PBS and lysed in 0.2 mol/L NaOH for ≥1 h at 37 °C. To determine the DNA concentration, 10 μL of lysate was transferred to a 96-well plate, to which 200 μL of freshly prepared assay buffer (1:1 mixture of 4 mol/L NaCl and 0.1 mol/L PO_4_ buffer, pH 7.4) supplemented with 0.1 μg/mL of the fluorescent DNA stain Hoechst 33342 (ImmunoChemistry Technologies, Bloomington, MN, USA). Fluorescence was read at an excitation wavelength of 340 ± 30 nm and an emission wavelength of 460 ± 40 nm using a microplate reader (BioTek). The DNA concentration per well was calculated using herring sperm DNA (Sigma) as standard. Experiments were performed at least twice.

### RNA extraction and quantification

Total RNA was isolated from snap-frozen liver samples using the MagNA Lyser system and the High Pure RNA Tissue Kit (both from Roche Applied Science). Purified RNA was reverse transcribed into cDNA using the Transcriptor First Strand cDNA Synthesis Kit (Roche Applied Science). Genomic data from the GenBank were used to design primer pairs spanning exon/intron boundaries of targets, which were synthesized by Biolegio (Nijmegen, the Netherlands). Alternatively, QuantiTect Primer Assays were obtained from Qiagen (Hilden, Germany). Primer sequences and GenBank accession numbers are listed in Supplementary Table S2. Quantitative reverse transcriptase polymerase chain reaction (qRT-PCR) was performed in duplicate or triplicate using a LightCycler 480 (Roche Applied Science) and SYBR Green probe (Roche Applied Science or SensiFAST, Bioline Reagents, London, UK). Melting curve analysis was performed to verify primer specificity. Data were analyzed according to Ruijter *et al.*^42^ and normalized to expression levels of *Gapdh*.

### Statistical analysis

Statistical analysis was performed in GraphPad Prism (GraphPad Software, La Jolla, CA, USA). Similarity of standard deviations was assessed with Bartlett’s test. When the distribution of data was non-Gaussian, data were transformed using logarithms and reciprocals. When the distribution of data remained non-Gaussian following transformation, the differences between ordinal variables were analyzed with a Kruskal-Wallis test and Dunn’s *post hoc* test for pair-wise comparison. For normally distributed data, an unpaired two-tailed *t*-test or a one-way analysis of variance (ANOVA) was used with Tukey’s *post hoc* test. A *p*-value of ≤0.05 was considered statistically significant.

## Additional Information

**How to cite this article**: Lionarons, D. A. *et al.* Simple steatosis sensitizes cholestatic rats to liver injury and dysregulates bile salt synthesis and transport. *Sci. Rep.* **6**, 31829; doi: 10.1038/srep31829 (2016).

## Supplementary Material

Supplementary Information

## Figures and Tables

**Table 1:** Plasma bile salt/acid composition.

Plasma	Sham	MCD	BDL	MCD+BDL
*CA*	3.8 ± 2.5	11.9 ± 6.2	5.5 ± 8.2	3.4 ± 1.2
*DCA*	3.4 ± 0.7	3.0 ± 0.8	3.3 ± 0.9	2.2 ± 0.2
*CDCA*	1.3 ± 0.7	2.3 ± 1.5^3,4^	0.6 ± 0.2	0.0 ± 0.0
*αMCA*	0.3 ± 0.2	0.9 ± 0.5	0.4 ± 0.7	0.3 ± 0.1
*ßMCA*	0.2 ± 0.2	1.5 ± 0.6	1.9 ± 2.6	1.2 ± 0.7
*ΩMCA*	0.0 ± 0.0	0.5 ± 0.3	0.8 ± 0.8^1^	0.2 ± 0.1
*TCA*	0.2 ± 0.5	0.0 ± 0.0	103.5 ± 65.2	677.2 ± 363.9^1,2^
*TDCA*	0.0 ± 0.0	0.2 ± 0.2	3.5 ± 3.2^1,2,4^	0.4 ± 0.5
*TCDCA*	0.0 ± 0.0	0.0 ± 0.0	9.7 ± 2.8	28.9 ± 13.4^1,2,3^
*TUDCA*	0.0 ± 0.0	0.0 ± 0.0	3.5 ± 5.3	10.0 ± 4.3^1,2,3^
*TαMCA*	0.0 ± 0.1	0.1 ± 0.0	12.9 ± 4.3^1^	25.8 ± 9.2^1,2^
*TßMCA*	0.0 ± 0.0	0.1 ± 0.1	235.7 ± 64.5^1,2^	454.2 ± 107.8^1,2,3^
*GCA*	0.6 ± 0.2	0.6 ± 0.3	4.3 ± 9.1	3.9 ± 1.1
*GDCA*	0.0 ± 0.0	0.0 ± 0.1	1.0 ± 0.6^1,2^	0.3 ± 0.5
*GCDCA*	0.0 ± 0.1	0.2 ± 0.2	1.3 ± 0.8^1^	2.1 ± 0.6^1,2^
*GUDCA*	0.0 ± 0.0	0.0 ± 0.0	0.0 ± 0.0	0.1 ± 0.1^1,2^

Concentrations of bile salt and bile acid species in plasma (μmol/L). Litocholate levels fell below the detection limit. Values represent means ± SD (*n* = 5–6); 1, *p* < 0.05 versus sham; 2, *p* < 0.05 versus MCD; 3, *p* < 0.05 versus BDL; 4, *p* < 0.05 versus MCD+BDL; CA, cholate; DCA, deoxycholate; CDCA, chenodeoxycholate; αMCA, α-muricholate; ßMCA, ß-muricholate; ΩMCA, Ω-muricholate; TCA, taurocholate; TDCA, taurodeoxycholate; TCDCA, taurochenodeoxycholate; TUDCA, tauroursodeoxycholate; TαMCA, tauro-α-muricholate; TßMCA, tauro-ß-muricholate; GCA, glycocholate; GDCA, glycodeoxycholate; GCDCA, glycochenodeoxycholate; GUDCA, glycoursodeoxycholate.

**Table 2:** Hepatic bile salt/acid composition.

Liver	Sham	MCD	BDL	MCD+BDL
*CA*	1.3 ± 1.3	1.4 ± 1.2	1.4 ± 1.6	0.7 ± 0.9
*ßMCA*	0.0 ± 0.0	0.1 ± 0.2	0.6 ± 0.7	0.3 ± 0.3
*TCA*	43.6 ± 18.3	34.2 ± 13.5	185.0 ± 86.6^1,2^	367.3 ± 130.7^1,2,3^
*TDCA*	14.1 ± 2.5	12.3 ± 3.0	8.8 ± 9.5	8.2 ± 4.0
*TCDCA*	10.0 ± 4.6	21.2 ± 4.9^1^	23.3 ± 9.9^1^	33.3 ± 14.1^1^
*TαMCA*	7.2 ± 1.1	7.3 ± 2.0	17.3 ± 6.8^1,2^	13.3 ± 5.7^1,2^
*TßMCA*	11.6 ± 2.3	7.6 ± 2.5	353.9 ± 111.6^1,2^	273.6 ± 122.2^1,2^
*GCA*	36.2 ± 7.4	20.5 ± 8.4	7.4 ± 16.1^1^	4.9 ± 0.8^1^
*GDCA*	7.0 ± 2.7	8.0 ± 5.0	0.6 ± 1.4^1,2^	0.0 ± 0.0^1,2^
*GCDCA*	8.4 ± 1.5	6.7 ± 2.4	5.4 ± 1.2^1^	4.6 ± 0.5^1^

Concentrations of bile salt and bile acid species in liver homogenates (μmol/kg liver). Litocholate, deoxycholate, chenodeoxycholate, α-muricholate, Ω-muricholate, tauroursodeoxycholate, and glycoursodeoxycholate levels fell below the detection limit. Values represent means ± SD (*n* = 5–6); 1, *p* < 0.05 versus sham; 2, *p* < 0.05 versus MCD; 3, *p* < 0.05 versus BDL; CA, cholate; ßMCA, ß-muricholate; TCA, taurocholate; TDCA, taurodeoxycholate; TCDCA, taurochenodeoxycholate; TαMCA, tauro-α-muricholate; TßMCA, tauro-ß-muricholate; GCA, glycocholate; GDCA, glycodeoxycholate; GCDCA, glycochenodeoxycholate.
