# Supplementary material for: Simple steatosis sensitizes cholestatic rats to liver injury and dysregulates bile salt synthesis and transport
Source: Sci Rep. 2016 Aug 18;6:31829. doi: 10.1038/srep31829 (PMC4989137; doi:10.1038/srep31829)
Supplement: Supplementary Information [file srep31829-s1.pdf]

SUPPLEMENTARY INFORMATION

Simple steatosis sensitizes cholestatic rats to liver injury and dysregulates bile salt synthesis and transport

Daniël A. Lionarons[1], Michal Heger[1], Rowan F. van Golen[1], Lindy K. Alles[1], Vincent A. van der Mark[1,2], Jaap J. Kloek[1], Dirk R. de Waart[2], Hendrik A. Marsman[1], Henny Rusch[3], Joanne Verheij[4], Ulrich Beuers[2], Coen C. Paulusma[2], Thomas M. van Gulik[1]

[1]Department of Experimental Surgery, [2]Tytgat Institute for Liver and Intestinal Research, [3]Laboratory Genetic Metabolic Diseases, and [4]Department of Pathology, Academic Medical Center, University of Amsterdam, Amsterdam, the Netherlands

Table of contents

Supplementary methods..3

Surgical procedures and tissue processing....................3

Measurement of bile salt composition............................4

CYP7A1 regulation assay 5

Supplementary Fig. S1...6

Supplementary Fig. S2...7

Supplementary Fig. S3...8

Supplementary Fig. S4...9

Supplementary Fig. S5...10

Supplementary Fig. S6...11

Supplementary Table S1..12

Supplementary Table S2..13

References..14

Supplementary methods

Surgical procedures and tissue processing

The animals were weighed before surgery. Surgery was performed under inhalation anesthesia comprising a mixture of oxygen:air (1:1 volume ratio, 2 L/min) and 2-4% isoflurane (Forene, Abbott Laboratories, Chicago, IL, USA). Animals received analgesia pre-operatively by subcutaneous administration of 250 μg/kg buprenorphine (Temgesic, Schering-Plough, Kenilworth, NJ, USA). Body temperature was maintained at 37.0 ± 0.2 °C. A midline laparotomy was performed and the common bile duct was mobilized. In animals undergoing BDL, the common bile duct was ligated proximally and distally relative to the liver, and the lumen was disconnected in between. In animals undergoing sham surgery, the bile duct was manipulated but not ligated and not disconnected. The abdomen was closed with dual layer sutures.

After 7 days the animals were weighed, anesthetized as described, and euthanized by exsanguination from the vena cava. Blood samples were collected in heparin and EDTA. Liver tissue was snap-frozen in liquid nitrogen and stored at -80 °C for subsequent RNA extraction and HPLC (left lateral and median lobe), and MPO activity, TNF-alpha, and IL-6 measurements (right lateral and median lobe). Samples were also fixed in 10% buffered formalin for histological analysis. A sample from the caudal lobe was weighed, desiccated at 37° C for 4 weeks, and weighed again to determine hepatic water content. Intergroup differences in hepatic water content, calculated by [1 – (dry weight/wet weight)] × 100%, were used as a measure of edema.

Measurement of bile salt composition

Individual bile salt/acid species were separated and quantified by reverse-phase HPLC [1,2]. Twenty µL of plasma was mixed with 5 volumes of acetonitrile to precipitate protein. For liver samples, ~100 mg tissue was sonicated in 5 volumes of H_2O for 30-90 s, followed by addition of 10 volumes of acetonitrile. Samples were centrifuged at 20,000 ×g for 10 min. Solvent was evaporated from supernatants, bile salts/acids were solubilized in 200 µL of 25% methanol, and 100 µL was applied to a Hypersil C18 HPLC column (internal diameter: 3 µm, length: 15 cm; Thermo Scientific, Waltham, MA, USA) operated at 20 °C. The starting eluent consisted of 6.8 mmol/L ammoniumformate (pH = 3.9), followed by linear gradient or isocratic elution with acetonitrile at the indicated concentration: 28% (1 min), 38% (13 min), 42% (19 min), 61% (20 min), 63% (25 min), 80% (28 min), 80% (31 min), and 0% (33 min). The flow rate was 0.8 mL/min. Detection was performed with an NQAD QT-500 (Quant Technologies, Blaine, MN, USA) and quantification was performed on the basis of calibration curves for all bile salt/acid species. We achieve good separation of the 17 relevant bile salt species present in rodents using this protocol, as demonstrated by a chromatogram (Supplementary Fig. S1). Total bile salt concentrations quantified using HPLC refer to the cumulative of all bile salts/acids, which were consistent with results from an enzyme cycling assay (Supplementary Fig. S4). The mean TβMCA/TCA ratio was calculated by averaging ratios from individual animals.

CYP7A1 regulation assay

HepG2-NTCP cells were cultured at 37 °C in humidified air with 5% CO_2 in Dulbecco's Modified Eagle's Medium (Lonza) supplemented with 10% fetal calf serum (Bodinco), 5 µg/mL insulin (Sigma), 50 µmol/L hydrocortisone hemisuccinate (Sigma), 100 IU/mL penicillin (Lonza), 0.1 mg/mL streptomycin (Lonza), and 2 mmol/L L-glutamine (Lonza). Cells were seeded in 24-well plates. When ~80% confluence was reached, cells were washed with PBS once and medium was changed to serum-free medium supplemented with 1% free fatty acid-free bovine serum albumin (Sigma) with or without addition of bile salts. Twenty-four hours after treatment, RNA was extracted using the RNeasy Mini Kit according to the manufacturer's instructions (Qiagen). Messenger RNA levels were quantified as described in the main article.

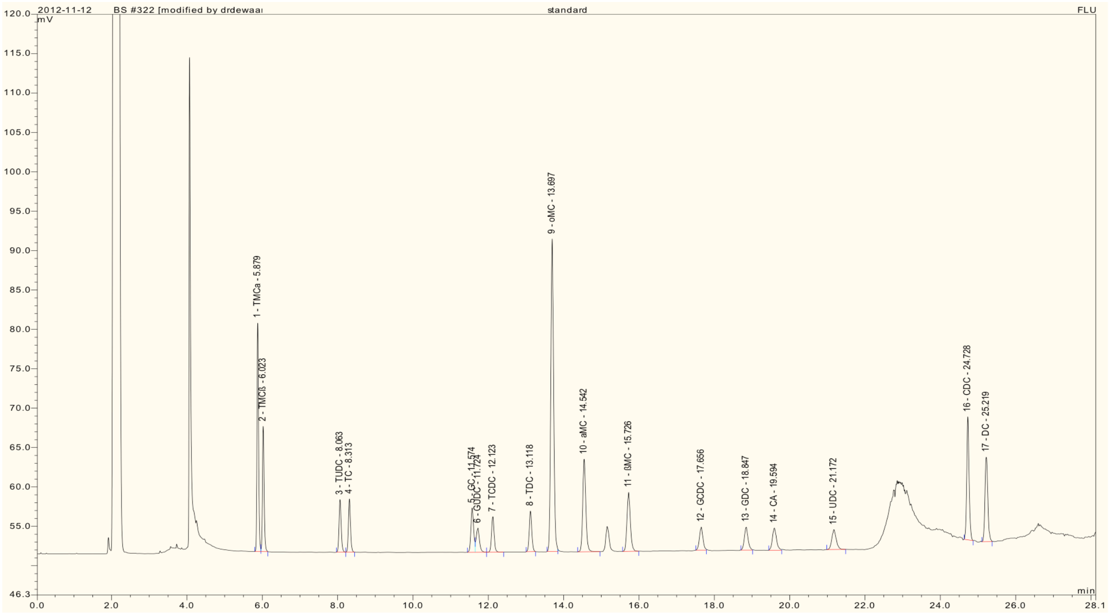

Supplementary Fig. S1. Chromatogram of 17 bile salt/acid species that comprise the rodent bile salt composition. These data demonstrate the complete separation of individual bile salt/acid species by HPLC. Litocholate appeared after 28 minutes and falls off this plot. Taurolitocholate eluted behind cholate as a separate peak (not shown). TMCa, tauro-α-muricholate; TMCß, tauro-ß-muricholate; TUDC, tauroursodeoxycholate; TC, taurocholate; GC, glycocholate; GUDC, glycoursodeoxycholate; TCDC, taurochenodeoxycholate; TDC, taurodeoxycholate; oMC, Ω-muricholate; aMC, α-muricholate; ßMC, ß-muricholate; GCDC, glycochenodeoxycholate; GDC, glycodeoxycholate; CA, cholate; UDC, ursodeoxycholate; CDC, chenodeoxycholate; DC, deoxycholate.

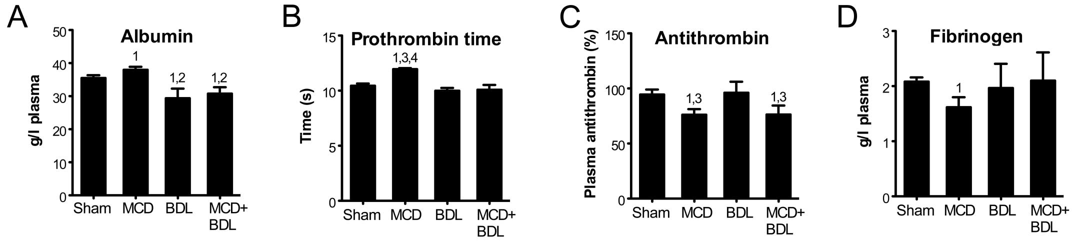

Supplementary Fig. S2. The hepatic synthesis function of animals with combined steatosis and cholestasis was not uniformly impaired compared to cholestasis alone. The liver synthesis function was assessed on the basis of (A) plasma albumin levels, (B) prothrombin time, (C) plasma antithrombin levels, and (D) plasma fibrinogen levels. Values represent mean ± SD for n = 5-6 per group. 1, $p < 0.05$ versus sham; 2, $p < 0.05$ versus MCD; 3, $p < 0.05$ versus BDL; 4, $p < 0.05$ versus MCD+BDL.

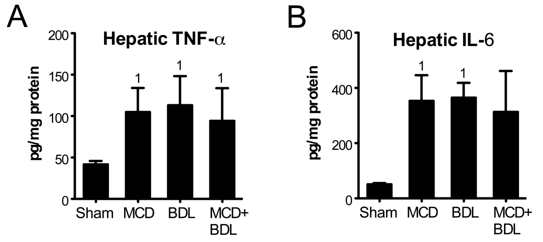

Supplementary Fig. S3. Hepatic levels of pro-inflammatory cytokines were comparable in rats with steatosis, cholestasis, and combined steatosis and cholestasis. Concentrations of TNF-α (A) and IL-6 (B) in liver homogenates were determined using an ELISA. Values represent means ± SD for n = 5-6 per group. 1, $p < 0.05$ versus sham.

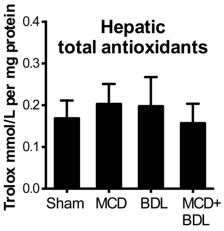

Supplementary Fig. S4. The hepatic total antioxidant capacity is similar in the liver of sham control rats, rats with steatosis, cholestasis, and combined steatosis and cholestasis. A total antioxidant capacity assay (Sigma) was performed as described previously [3]. There were no statistically significant differences detected between the 4 groups (p = 0.42). Values represent means ± SD for n = 5-6 samples.

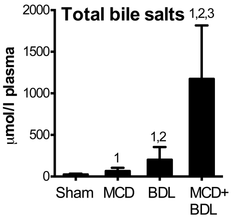

Supplementary Fig. S5. Measurement of plasma total bile salts with an enzyme cycling assay. The data are in line with HPLC results shown in Fig. 3B, indicating that plasma total bile salt levels were substantially elevated in animals with combined steatosis and cholestasis versus cholestasis ($p < 0.001$). Values represent means ± SD for $n = 5\text{-}6$ samples. 1, $p < 0.05$ versus sham; 2, $p < 0.05$ versus MCD; 3, $p < 0.05$ versus BDL.

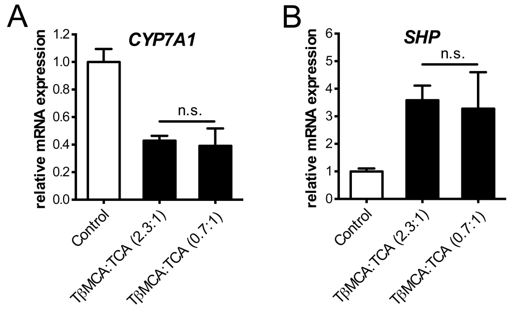

Supplementary Fig. S6. Gene expression of *CYP7A1* and *SHP* in HepG2 cells treated with TβMCA and TCA. HepG2-NTCP cells were incubated with bile salts at a total concentration of 100 µmol/L with a differential molar ratio for 24 h. *CYP7A1* (A) and *SHP* (B) mRNA was quantified using qRT-PCR. Values represent means ± SD (4 biological replicates from 2 independent experiments); n.s., not significant ($p > 0.05$).

Supplementary Table S1. NAFLD activity score.

NASH activity score (range)	Sham	MCD	BDL	MCD+BDL
Steatosis (0-3)	0 (0-0)	3 (3-3)	0 (0-0)	0 (0-1)
Lobular inflammation (0-3)	1 (1-1)	1 (1-1)	1 (1-1)	1 (1-1)
Hepatocellular ballooning (0-2)	0 (0-0)	0 (0-0)	0 (0-0)	0 (0-1)
Total score (0-8)	1 (1-1)	4 (4-4)	1 (1-1)	2 (1-2)

NAFLD activity scores demonstrate that none of the animals met the histological criteria for NASH diagnosis, which requires a total score ≥ 5. The validated scoring system as described by Kleiner et al. [4] was applied. Scores are shown as median (range) for *n* = 5-6 animals per group.

Supplementary Table S2. Primers used for qRT-PCR.

Target	Species	Accession no.	Direction	Sequence
Gapdh	Rat	NM_017008.3	Forward	acatcatccctgcatccactgg
			Reverse	catacttggcaggtttctccagg
Cyp7a1	Rat	NM_012942.1	Forward	gcagcctctgaagaagtgagtgg
			Reverse	gatgctgtctagtaccggcagg
Shp	Rat	NM_057133.1	Forward	gctagaggaacccaacagtggt
			Reverse	cctggcacatctgggttgaaga
Fxr	Rat	NM_021745.1	Forward	gtcatcctctctccagacagaca
			Reverse	ggttgaatgtccggagttctgtc
Ntcp	Rat	NM_017047.1	Forward	tgaacctcagcatcgtgatgacc
			Reverse	ggacgatccctatggtgcaagg
Oatp1a1	Rat	NM_017111.1	Forward	ctggaaccaacatggtgtttcagg
			Reverse	gtactgcagcctgttggcacac
Oatp1a4	Rat	NM_131906.1	Forward	ctcttatgaaggggttcagcacc
			Reverse	caaggcaggctgacatgtatgc
Oatp1b2	Rat	NM_031650.2	Forward	cgagcactaggagggattctagc
			Reverse	gcctacatgcaccacgttttcc
Bsep	Rat	NM_031760.1	Forward	ctctgctttgccttttcccagg
			Reverse	agagaccaccctgaaaacgtgg
Mrp2	Rat	NM_012833.1	Forward	gagtctgaggatgaatctcgacc
			Reverse	tgccctatgctcaggttgtcacc
Mrp3	Rat	NM_080581.1	Forward	atgctggccaaaatgcggttgc
			Reverse	ccaggagcccttgcagtattcc
Mrp4	Rat	NM_133411.1	Forward	gttctggcaaagaccttggatgc
			Reverse	ccgtgtattcaatcaccctctcc
GAPDH	Human	NM_002046.5	Forward	catcaccatcttccaggag
			Reverse	tgcccacagccttggcagc
CYP7A1	Human	NM_000780	Forward	N.A. (cat. no. QT00005726)
			Reverse	
SHP	Human	NM_021969	Forward	N.A. (cat. no. QT00061460)
			Reverse	

Sequences were designed using genomic and cDNA data obtained from GenBank (NCBI). Human *CYP7A1* and *SHP* primers were from Qiagen (QuantiTect Primer Assay). Sequences that were not available from the manufacturer are designated as N.A.

References

[1] Kunne, C. et al. Defective bile salt biosynthesis and hydroxylation in mice with reduced cytochrome P450 activity. *Hepatology* **57**, 1509-1517 (2013).

[2] Slijepcevic, D. et al. Impaired uptake of conjugated bile acids and hepatitis b virus pres1-binding in na(+) -taurocholate cotransporting polypeptide knockout mice. *Hepatology* **62**, 207-219 (2015).

[3] Kloek, J. J. et al. Cholestasis is associated with hepatic microvascular dysfunction and aberrant energy metabolism before and during ischemia-reperfusion. *Antioxid. Redox Signal.* **17**, 1109-1123 (2012).

[4] Kleiner, D. E. et al. Design and validation of a histological scoring system for nonalcoholic fatty liver disease. *Hepatology* **41**, 1313-1321 (2005).
